# Factors associated with aortic valve stenosis in Japanese patients with end-stage kidney disease

**DOI:** 10.1186/s12882-022-02758-y

**Published:** 2022-04-02

**Authors:** Yuji Sasakawa, Naoki Okamoto, Maya Fujii, Jyoichiro Kato, Yukio Yuzawa, Daijo Inaguma

**Affiliations:** 1 Department of Internal Medicine, Fujita Health University Bantane Hospital, Aichi, Japan; 2 Department of Nephrology, Fujita Health University, Aichi, Japan

**Keywords:** Dialysis, Aortic valve stenosis, Aortic valve calcification, Phosphorus

## Abstract

**Background:**

Aortic valve stenosis (AS) has a high prevalence and poor prognosis in patients who receive maintenance dialysis. However, few large-scale observational studies in Japan have investigated patients with AS who underwent dialysis. In this study, we investigated the prevalence and factors associated with AS in Japanese patients who underwent dialysis.

**Methods:**

In this cross-sectional analysis, we enrolled patients who underwent dialysis and transthoracic echocardiography between July 1, 2017 and June 30, 2018. Patients with a maximum aortic jet velocity (Vmax) ≥ 2.0 m/s, pressure gradient (PG) between the left ventricle and ascending aorta (mean PG) ≥ 20 mmHg, or aortic valve area (AVA) ≤ 1.0 cm^2^ were categorized into the AS group (G1). Patients with Vmax ≥ 3.0 m/s, mean PG ≥ 20 mmHg, or AVA ≤ 1.0 cm^2^ were categorized into the moderate and severe AS groups (G2). We performed multivariate logistic regression analysis and compared G1 and G2 with the non-AS group to determine the risk factors for AS. We also investigated the risk factors for aortic valve calcification, which is a pre-stage for AS.

**Results:**

Of the 2,786 patients investigated, 555 (20.0%) and 193 (6.9%) were categorized into G1 and G2, respectively. Multivariate logistic regression analysis revealed that age, long-term dialysis, and elevated serum phosphorus levels were associated with AS in both the groups (*p* < 0.05). These factors were converted into ordinal categories, and a multivariate logistic regression analysis was performed. Patients with serum phosphorus levels measuring 5.0–5.9 mg/dL and > 6.0 mg/dL showed a higher risk of AS than those with serum phosphorus levels measuring < 4.0 mg/dL (odds ratio 2.24, *p* = 0.01 and odds ratio 2.66, *p* = 0.005, respectively). Aortic valve calcification was associated with age, long-term dialysis, diabetes mellitus, administration of vitamin D receptor activators, elevated serum calcium levels, and anemia (*p* < 0.05 for all).

**Conclusions:**

Patients on dialysis showed a high prevalence of AS, which was associated with age, long-term dialysis, and elevated serum phosphorus levels.

**Trial registration:**

UMIN000026756, registered on March 29, 2017.

**Supplementary Information:**

The online version contains supplementary material available at 10.1186/s12882-022-02758-y.

## Background

Chronic kidney disease (CKD) is a significant risk factor for cardiovascular disease (CVD) [1–5]. The prevalence of CVD, particularly aortic valve stenosis (AS), ranges from 13 to 25% in patients on dialysis, [6, 7] which is higher than the prevalence (2.3%–4.3%) in the general elderly population [8, 9]. Previous clinical studies have reported that AS in patients on dialysis is characterized by a rapid decrease in aortic valve area (AVA) and rapid progression of valve calcification [10, 11]. Additionally, survival rates are lower in AS patients with CKD than in those without [12, 13].

Age-induced changes in the aortic valve, leading to sclerosis and calcification, [14] have replaced rheumatic fever as the primary cause of AS [15]. Morphological changes secondary to mechanical stress, endothelial injury, inflammation, and valve hemorrhage [16] can lead to aortic valve leaflet thickening, fibrosis, and calcification, eventually causing stenosis. However, the mechanisms underlying these changes remain unclear. Regarding the risk factors, a few studies have reported that age, male sex, hypertension, dyslipidemia, and smoking are associated with valve degeneration [9, 17, 18]. Other studies have reported that hypertension, dyslipidemia, diabetes mellitus, and renal insufficiency are not associated with AS progression [19–21].

The prevalence of hypertension, diabetes mellitus, and mineral and bone disorders (MBD) is higher in patients on dialysis compared to the general population. These conditions are associated with arteriosclerosis and CVD [22–28]. The factors associated with AS in patients on dialysis have not been investigated by a large-scale cohort study. Therefore, we conducted a multicenter prospective cohort study to investigate the factors associated with AS, the prevalence, and prognosis of patients with AS who have had dialysis. In this study, only the results of the cross-sectional analysis of the included patients at baseline in the multicenter prospective cohort study were reported.

## Materials and methods

### Study population

The study included patients aged ≥ 20 years who underwent outpatient maintenance dialysis for > 1 year across 58 hospitals in the Tokai region of Japan. The baseline was selected as the time of transthoracic echocardiography (TTE) performed between July 1, 2017 and June 30, 2018. Patients with a history of aortic valve surgery and those who refused to participate in the study were excluded. 2,916 patients were enrolled and the following data was recorded: Patients’ background, history, comorbidities, medications, laboratory data, chest radiography findings, electrocardiogram tracings, and TTE images. Out of the 2,916 patients enrolled in the study, 130 patients who met the following criteria were excluded: duration of dialysis < 1 year, unknown initiation of dialysis, history of aortic valve surgery, or TTE performed outside the observation period.

### Definition of aortic stenosis and aortic valve calcification

**Fig. 1:**
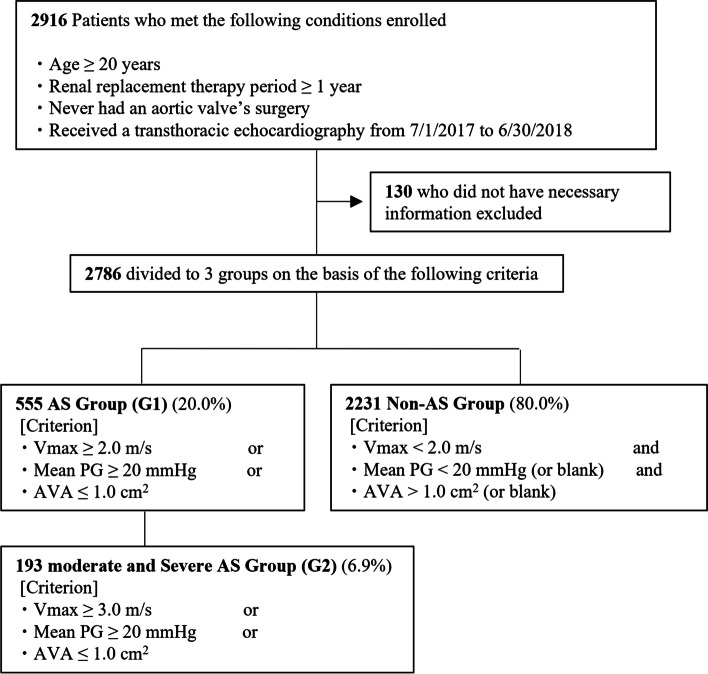
A flow diagram of the present study**.** Abbreviations: Vmax, Velocity max; PG, pressure gradient; AVA, aortic valve area

All the patients underwent a TTE to measure the parameters associated with AS and to evaluate for aortic valve calcification. The maximum aortic jet velocity (Vmax), pressure gradient (PG) between the left ventricle and ascending aorta (mean PG), and the AVA was recorded to evaluate for AS. Aortic stenosis was defined based on the 2014 American Heart Association/American College of Cardiology Guidelines for the Management of Patients with Valvular Heart Disease. It was defined as (a) Vmax ≥ 2.0 m/s, (b) mean PG ≥ 20 mmHg, or (c) AVA ≤ 1.0 cm^2^. Patients who met any of the criteria were categorized into the AS group (G1); the rest were categorized into the non-AS group. Among the patients in G1, the moderate and severe AS groups were categorized as G2, with the Vmax criterion changed from ≥ 2.0 m/s to ≥ 3.0 m/s without any change in the mean PG and AVA criteria. Fig. 1 shows the patient flowchart.

### Statistical analysis

Baseline data is presented as means (standard deviation), medians (interquartile range), or percentages for categorical measures. Fisher's exact test for nominal variables and the Mann–Whitney U test for continuous variables was used to compare the baseline data between G1 and the non-AS group and between G2 and the non-AS group. Factors associated with AS on univariate analysis were subjected to multivariate logistic regression analysis along with the factors associated with MBD. The following 14 variables were subjected to multivariate logistic regression analysis: sex, age, duration of dialysis, nephrosclerosis as the primary contributor to CKD, diabetes mellitus as a comorbidity, calcimimetic use, vitamin D receptor activator (VDRA) administration, phosphate binder use, serum albumin level, serum corrected calcium level, serum phosphorus level, serum intact parathyroid hormone level, serum C-reactive protein level, and serum hemoglobin level. Multivariate logistic regression analysis was performed with these 14 variables, and age, dialysis duration, and serum phosphorus levels that showed significant differences were converted into ordinal categories. Patients were categorized on the basis of age into the following groups: < 60, 60–69, 70–79, and ≥ 80 years. Multivariate logistic regression analysis was performed on these age ordinal categories and 13 other factors. Similarly, Patients were categorized into the following groups on the basis of the duration of dialysis and serum phosphorus levels: < 5, 5–9, 10–14, and ≥ 5 years and < 4.0, 4.0–4.9, 5.0–5.9, and ≥ 6.0 mg/dL, respectively.

Patients were further categorized into those with and without aortic valve calcification (a pre-stage of AS), and a multivariate logistic regression analysis was performed. The Mann–Whitney U test was used to compare the Vmax, mean PG, and AVA between patients with and without aortic valve calcification.

All statistical analyses were performed using the R software (The R Foundation for Statistical Computing, Vienna, Austria). All values were two-sided, and *p* < 0.05 was considered statistically significant.

### Ethics approval and consent to participate

This study was performed in accordance with the Ethics of Clinical Research (The Helsinki Declaration) and was approved by the Fujita Health University Center for Clinical Trial and Research Support (approval numbers: HM16–373). All patients included in this study provided informed consent after the purpose of the study was explained to them. This study was registered in the Clinical Trial Registry (UMIN 000,026,756) on April 1, 2017.

## Results

### Baseline patient characteristics

In total, 2,786 patients were enrolled in the study; 555 patients (20.0%) were categorized into G1 and 2,231 (80.0%) into the non-AS group. Out of the 555 patients in G1, 514 (92.6%) showed Vmax ≥ 2.0 m/s, 107 (19.3%) showed mean PG ≥ 20 mmHg, and 102 (18.6%) showed AVA ≤ 1.0 cm^2^. Based on these findings, the moderate and severe AS groups were categorized as G2, with the Vmax criterion changed from ≥ 2.0 m/s to ≥ 3.0 m/s without any change in the mean PG and AVA criteria. Among patients in G1, 193 (6.9%) were categorized as G2. Out of the 193 patients, 98 (50.8%) showed Vmax ≥ 3.0 m/s, 107 (55.4%) showed mean PG ≥ 20 mmHg, and 102 (52.8%) showed AVA ≤ 1.0 cm^2^. Table 1 shows a comparison of the baseline characteristics between G1 and the non-AS group and between G2 and the non-AS group. The median ages of G1, G2, and the non-AS groups were 75, 77, and 68 years, respectively. The median hemodialysis durations in G1, G2, and the non-AS groups were 8.0, 7.4, and 6.2 years, respectively. The prevalence of diabetes mellitus in G1, G2, and the non-AS groups were 36.0%, 39.9%, and 46.0%, respectively. No significant differences were observed in the mean serum phosphorus and corrected serum calcium levels in the groups. According to the TTE results, the percentages of aortic valve calcification in G1 and G2 were 80.7% and 83.8%, respectively, which were higher than the percentage in the non-AS group (53.3%).

**Table 1:** Comparison of baseline characteristics between G1 and the non-AS group and between G2 and the non-AS group

	**non-AS group**	**G1**	***p* value**	**G2**	***p* value**
**Basic Characteristics**					
Male, %	67.0	63.6	0.13	59.6	0.04
Age, years	68 (59–75)	75 (68–82)	< 0.001	77 (70–83)	< 0.001
Duration of dialysis, years	6.2 (3.1–12.4)	8.0 (3.8–15.1)	< 0.001	7.4 (4.0–14.8)	0.005
**Primary Disease**					
Nephrosclerosis, %	11.9	17.3	0.001	16.6	0.07
Diabetic nephropathy, %	39.8	29.5	< 0.001	31.6	0.03
Chronic glomerulonephritis, %	19.5	23.8	0.03	23.8	0.16
**Comorbidity**					
Diabetes mellitus, %	46.0	36.0	< 0.001	39.9	0.11
Admission due to HF < 1 year, %	2.0	5.2	< 0.001	8.3	< 0.001
Percutaneous coronary intervention, %	10.8	15.5	0.003	16.1	0.03
Coronary artery bypass grafting, %	4.7	6.7	0.07	9.3	0.01
Aortic disease, %	4.8	10.8	< 0.001	14.0	< 0.001
**Usage rate of medicine related to MBD**					
Vitamin D receptor activators, %	81.3	79.6	0.40	78.2	0.29
Calcimimetics, %	34.9	35.5	0.80	38.3	0.35
Phosphate binders, %	84.7	78.6	< 0.001	77.2	0.01
**Blood Test**					
Corrected Calcium, mg/dl	9.1 ± 0.6	9.1 ± 0.6	0.16	9.1 ± 0.7	0.55
Phosphorus, mg/dl	5.3 ± 1.3	5.2 ± 1.2	0.76	5.2 ± 1.2	0.59
Intact PTH, pg/ml	132 (80–193)	122 (76–185)	0.05	118 (76–162)	0.04
Albumin, g/dl	3.6 ± 0.4	3.5 ± 0.4	< 0.001	3.4 ± 0.37	< 0.001
C-reactive protein, mg/dl	0.12 (0.05–0.37)	0.12 (0.06–0.43)	0.15	0.11 (0.06–0.55)	0.15
Hemoglobin, g/dl	11.1 ± 1.1	10.9 ± 1.2	< 0.001	10.8 ± 1.1	0.002
**Chest radiography**					
Cardiothoracic ratio, %	50.2 ± 5.1	52.2 ± 5.0	< 0.001	53.0 ± 5.0	< 0.001
Aortic calcification, %	57.0	70.4	< 0.001	73.3	< 0.001
**Electrocardiogram**					
Atrial fibrillation, %	5.7	9.6	0.002	12.0	0.002
Heart rate, /min	74 ± 13	74 ± 13	0.91	75 ± 13	0.63
**Transthoracic Echocardiography**					
Left atrial diameter, mm	36.6 ± 6.5	38.5 ± 6.4	< 0.001	39.1 ± 5.9	< 0.001
LVDd, mm	46.9 ± 6.8	46.9 ± 6.1	0.63	46.2 ± 6.4	0.26
LVDs, mm	30.6 ± 6.4	30.5 ± 6.0	0.96	30.1 ± 6.6	0.17
Left ventricular ejection fraction, %	63.4 ± 9.7	63.5 ± 10.0	0.52	63.5 ± 11.3	0.37
Aortic valve calcification, %	53.3	80.7	< 0.001	83.8	< 0.001
Mean pressure gradient, mmHg	5.1 ± 2.7	15.5 ± 9.6	< 0.001	23.3 ± 11.8	< 0.001
Aortic valve area, cm2	2.51 ± 0.77	1.56 ± 0.62	< 0.001	1.08 ± 0.46	< 0.001
Velocity max, m/s	1.43 ± 0.28	2.52 ± 0.62	< 0.001	2.96 ± 0.87	< 0.001

Data are presented as the mean ± standard deviation or percentages. Age, dialysis duration, intact PTH, and C-reactive protein are expressed as the median (interquartile range)

*Abbreviations*: Admission due to HF < 1 year, admission due to heart failure within 1 year before echocardiography; *LVDd* Left ventricular end-diastolic diameter, *LVDs* Left ventricular end-systolic diameter

### Multivariate logistic regression analysis for aortic stenosis

We performed a multivariate logistic regression analysis to compare G1 and G2 with the non-AS group (Table 2). Comparison between G1 and the non-AS group showed that age, long-term dialysis, and elevated serum phosphorus levels were associated with AS (adjusted odds ratio [aOR] 1.93, 95% confidence interval [CI] 1.71–2.19, *p* < 0.001; aOR 1.41, 95% CI 1.21–1.64, *p* < 0.001; aOR 1.16, 95% CI 1.06–1.28, *p* = 0.001, respectively). Comparison between G2 and the non-AS group showed similar tendencies in these three variables (aOR 2.51, 95% CI 2.02–3.12, *p* < 0.001; aOR 1.35, 95% CI 1.06–1.71, *p* = 0.01; aOR 1.24, 95% CI 1.07–1.44, *p* = 0.005, respectively). In contrast, nephrosclerosis as a contributor to CKD was not associated with AS. Diabetes mellitus as a comorbidity was associated with AS when G1 was compared to the non-AS group (aOR 0.74, 95% CI 0.58–0.95, *p* = 0.02); however, no significant difference was observed between G2 and the non-AS group (aOR 0.95, 95% CI 0.64–1.40, *p* = 0.79).

**Table 2:** Adjusted odds ratio for aortic valve stenosis by multivariate logistic regression analysis

	**G1**		**G2**	
	aOR (95% CI)	*p* value	aOR (95% CI)	*p* value
Male	1.02 (0.81–1.29)	0.86	0.94 (0.65–1.37)	0.75
Age (per 10 years)	1.93 (1.71–2.19)	< 0.001	2.51 (2.02–3.12)	< 0.001
Duration of dialysis (per 10 years)	1.41 (1.21–1.64)	< 0.001	1.35 (1.06–1.71)	0.01
Nephrosclerosis as the primary contributor to CKD	0.94 (0.67–1.32)	0.72	0.88 (0.52–1.50)	0.65
Diabetes mellitus comorbidity	0.74 (0.58–0.95)	0.02	0.95 (0.64–1.40)	0.79
Calcimimetic use	1.16 (0.91–1.48)	0.24	1.59 (1.07–2.34)	0.02
VDRA administration	0.94 (0.70–1.25)	0.66	0.91 (0.58–1.44)	0.69
Phosphate binder use	0.87 (0.65–1.17)	0.35	0.86 (0.55–1.35)	0.52
Corrected Calcium (per 1 mg/dl)	1.27 (1.04–1.54)	0.02	1.17 (0.86–1.59)	0.31
Phosphorus (per 1 mg/dl)	1.16 (1.06–1.28)	0.001	1.24 (1.07–1.44)	0.005
Intact PTH (per 10 pg/ml)	1.00 (0.99–1.01)	0.41	1.00 (0.98–1.02)	0.91
Albumin (per 1 g/dl)	1.51 (1.05–2.17)	0.03	1.04 (0.58–1.84)	0.91
C-reactive protein (per 1 mg/dl)	0.98 (0.89–1.08)	0.67	1.02 (0.91–1.14)	0.77
Hemoglobin (per 1 g/dl)	0.85 (0.76–0.95)	0.003	0.86 (0.72–1.03)	0.10

Multivariate logistic regression analysis were perfomed to compare G1 and G2 with the non-AS group

*Abbreviations*: *aOR* adjusted Odds Ratio, *CI* Confidence interval, *CKD* Chronic kidney disease, *VDRA* Vitamin D receptor activator

Multivariate logistic regression analysis for aortic stenosis using ordinal categories.

**Fig. 2:**
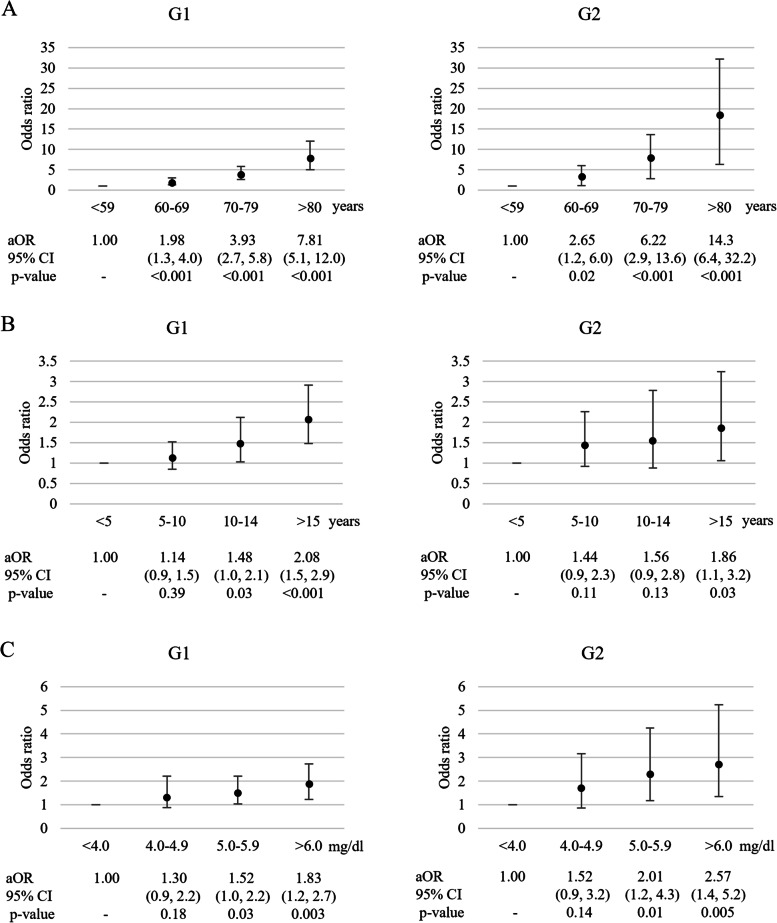
Adjusted odds ratio (aOR) for AS between the categories of age, duration of dialysis, and serum phosphorus level. The graph on the left was a comparison between G1 and the non-AS group, and the graph on the right was a comparison between G2 and the non-AS group. The multivariate analysis model included the sex, age, duration of dialysis, nephrosclerosis as the primary contributor to CKD, diabetes mellitus as a comorbidity, calcimimetic use, vitamin D receptor activators administration, phosphate binder use, serum albumin level, serum corrected calcium level, serum phosphorus level, serum intact parathyroid hormone level, serum C-reactive protein level, and serum hemoglobin level as variables. A: aOR for AS between the categories of age. B: aOR for AS between the categories of duration of dialysis. C: aOR for AS between the categories of serum phosphorus levels

The aOR for AS was determined on the basis of age, duration of dialysis, and serum phosphorus levels (Figs. 2A, 2B, and 2C). The aOR in the AS group tended to increase with an increase in the duration of dialysis. In G2, the aOR of > 15 years of dialysis was 1.86-fold greater (95% CI 1.06–3.24, *p* = 0.03) than that of < 5 years of dialysis. The aOR increased with an increase in serum phosphorus levels. The aORs of the serum phosphorus levels in the 5.0–5.9 and > 6.0 mg/dL groups were 1.52 and 1.83-fold higher and 2.24 and 2.66-fold higher in G1 and G2, respectively, compared to serum phosphorus levels < 4.0 mg/dL.

### Characteristics of patients with aortic valve calcification

Table 3 shows the results of the multivariate logistic regression analysis for aortic valve calcification. Aortic valve calcification was associated with age, long-term dialysis, diabetes mellitus, VDRA administration, elevated serum calcium levels, and anemia but not with elevated serum phosphorus levels. The mean PG, AVA, and Vmax values were compared in patients with and without aortic valve calcification (Table 4). Significantly higher Vmax and mean PG values and significantly lower AVA values were observed in patients with aortic valve calcification.

**Table 3:** Adjusted odds ratio for aortic valve calcification by multivariate logistic regression analysis

	Aortic valve calcification	
	OR (95% CI)	*p* value
Male	0.93 (0.75–1.16)	0.53
Age (per 10 years)	1.70 (1.54–1.88)	< 0.001
Duration of dialysis (per 10 years)	1.42 (1.22–1.64)	< 0.001
Nephrosclerosis as the primary contributor to CKD	0.88 (0.69–1.06)	0.43
Diabetes mellitus comorbidity	1.53 (1.23–1.90)	< 0.001
Calcimimetic use	0.86 (0.69–1.06)	0.16
VDRA administration	0.70 (0.54–0.91)	0.007
Phosphate binder use	0.92 (0.69–1.23)	0.57
Corrected Calcium (per 1 mg/dl)	1.30 (1.09–1.54)	0.004
Phosphorus (per 1 mg/dl)	1.03 (0.95–1.12)	0.46
Intact PTH (per 10 pg/ml)	1.00 (0.99–1.01)	0.56
Albumin (per 1 g/dl)	1.19 (0.87–1.63)	0.28
C-reactive protein (per 1 mg/dl)	0.98 (0.90–1.07)	0.65
Hemoglobin (per 1 g/dl)	0.89 (0.81–0.98)	0.02

**Table 4:** Comparison of parameters with and without aortic valve calcification

	**With** aortic valve calcification	**Without** aortic valve calcification	I value
Velocity max, m/s	1.78 ± 0.64	1.51 ± 0.50	< 0.001
Mean pressure gradient, mmHg	8.3 ± 7.9	6.3 ± 4.8	< 0.001
Aortic valve area, cm^2^	2.19 ± 0.79	2.50 ± 0.90	< 0.001

Analyses were performed using Mann–Whitney U test

## Discussion

This study showed that the prevalence of AS in patients on dialysis was as high as 20.0%, which is similar to the findings of previous reports [6, 7]. A close association between AS and aortic valve calcification was observed. Age was strongly associated with AS in patients on dialysis, as well as in the general population. Notably, hyperphosphatemia and long-term dialysis were associated with AS.

Hyperphosphatemia is seen in many patients on dialysis. Based on the United States Renal Data System statistics published in 2018, 38.1% of patients on dialysis in the USA had serum phosphorus levels ≥ 5.5 mg/dL. Based on the data published by the Japanese Society for Dialysis Therapy in 2019, 53.9% of patients on dialysis in Japan had serum phosphorus levels ≥ 5.0 mg/dL. Hyperphosphatemia promotes calcification of vascular smooth muscle cells and promotes CVD, which is associated with a high mortality in patients with CKD [29–37]. Phosphorus is considered a major contributor to calcification, in both blood vessels and the aortic valve [38]. Husseini et al. reported that SLC20A1/Pit1, a phosphorus transporter associated with calcification in blood vessels, is also expressed in calcified aortic valves [39]. In two different observational studies that included approximately 100 patients who underwent dialysis, Tarrass et al. and Petrović et al. reported that phosphorus was associated with aortic valve calcification on the basis of univariate and multivariate analyses results, respectively [40, 41]. However, the association between hyperphosphatemia and AS has not been studied. In this study, an elevated serum phosphorus level was not associated with aortic valve calcification but was associated with AS. In contrast, an elevated serum calcium level was not associated with AS but was associated with aortic valve calcification. Based on these results, MBD, including hyperphosphatemia and hypercalcemia, may be associated with aortic valve calcification and AS. Interestingly, serum phosphorus levels ≥ 5.0 mg/dL (lower than the upper limit of 6.0 mg/dL established by Japanese guidelines) were associated with AS. The findings may be useful to establish target values for serum phosphorus levels.

This study showed that long-term dialysis was associated with both AS and aortic valve calcification on multivariate analysis, which included age and MBD-related factors as variables. Long-term dialysis involves various factors, such as fluid retention, chronic inflammation, and uremic toxins, that affect the cardiovascular system; therefore, it may be difficult to identify the factors that contribute to AS [42–47].

### Limitations

The following are the limitations of this study. (i) Patients with a history of aortic valve surgery were excluded; therefore, a serious risk factor associated with AS may have been disregarded. (ii) This study was a cross-sectional analysis; therefore, the results cannot demonstrate causality. (iii) The accuracy of TTE was unlikely to be consistent across patients in this multicenter study. Aortic valve calcification was based on the subjective interpretation of echocardiography findings by technicians at each facility. (iv) Data were not collected on whether the patients had bicuspid aortic valves, which is one of the major causes of aortic stenosis. This may have affected the results of this study. (v) The percentages of missing values for Vmax, mean PG, and AVA were 12.2%, 25.4%, and 20.9%, respectively. Although one or more of these factors were measured in all patients, missing data may potentially lead to misclassification of the AS groups. (vi) The multivariate logistic regression analysis was performed by complete cases analysis. This may have affected the results.

## Conclusion

Patients who received maintenance dialysis showed a high prevalence of AS, which was associated with age, hyperphosphatemia, and duration of dialysis.

## Supplementary Information

**Additional file 1.**

## Data Availability

The dataset analyzed in the present study is presented in the additional file and available (title of data: dataset 1). The dataset included the patient profiles, comorbidities, medications, laboratory data, ECG, chest X-ray, and TTE.

## Abbreviations

CKD: Chronic kidney disease
CVD: Cardiovascular disease
AS: Aortic valve stenosis
AVA: Aortic valve area
MBD: Mineral and Bone Disorder
TTE: Transthoracic echocardiography
PG: Pressure gradient
Vmax: Maximum aortic jet velocity
MOR: Multivariate-adjusted odds ratio
CI: Confidence interval
VDRA: Vitamin D receptor activators

## References

[1] Go AS, Chertow GM, Fan D, McCulloch CE, Hsu CY (2004). Chronic kidney disease and the risks of death, cardiovascular events, and hospitalization. N Engl J Med.

[2] Kottgen A, Russell SD, Loehr LR, Crainiceanu CM, Rosamond WD, Chang PP, Chambless LE, Coresh J (2007). Reduced kidney function as a risk factor for incident heart failure: the atherosclerosis risk in communities (ARIC) study. J Am Soc Nephrol.

[3] Anavekar NS, McMurray JJ, Velazquez EJ, Solomon SD, Kober L, Rouleau JL, White HD, Nordlander R, Maggioni A, Dickstein K, Zelenkofske S, Leimberger JD, Califf RM, Pfeffer MA (2004). Relation between renal dysfunction and cardiovascular outcomes after myocardial infarction. N Engl J Med.

[4] Wanner C, Amann K, Shoji T (2016). The heart and vascular system in dialysis. Lancet.

[5] Cabrera CS, Lee AS, Olsson M, Schnecke V, Westman K, Lind M, Greasley PJ, Skrtic S (2020). Impact of CKD progression on cardiovascular disease risk in a contemporary UK cohort of individuals with diabetes. Kidney Int Rep.

[6] Straumann E, Meyer B, Misteli M, Blumberg A, Jenzer HR (1992). Aortic and mitral valve disease in patients with end stage renal failure on long-term haemodialysis. Br Heart J.

[7] Schönenberger A, Winkelspecht B, Köhler H, Girndt M (2004). High prevalence of aortic valve alterations in haemodialysis patients is associated with signs of chronic inflammation. Nephron Clin Pract.

[8] Otto CM, Lind BK, Kitzman DW, Gersh BJ, Siscovick DS (1999). Association of aortic-valve sclerosis with cardiovascular mortality and morbidity in the elderly. N Engl J Med.

[9] Stewart BF, Siscovick D, Lind BK, Gardin JM, Gottdiener JS, Smith VE, Kitzman DW, Otto CM (1997). Clinical factors associated with calcific aortic valve disease. cardiovascular health study. J Am Coll Cardiol.

[10] Perkovic V, Hunt D, Griffin SV, du Plessis M, Becker GJ (2003). Accelerated progression of calcific aortic stenosis in dialysis patients. Nephron Clin Pract.

[11] Ohara T, Hashimoto Y, Matsumura A, Suzuki M, Isobe M (2005). Accelerated progression and morbidity in patients with aortic stenosis on chronic dialysis. Circ J.

[12] Samad Z, Sivak JA, Phelan M, Schulte PJ, Patel U, Velazquez EJ (2017). Prevalence and outcomes of left-sided valvular heart disease associated with chronic kidney disease. J Am Heart Assoc.

[13] Zentner D, Hunt D, Chan W, Barzi F, Grigg L, Perkovic V (2011). Prospective evaluation of aortic stenosis in end-stage kidney disease: a more fulminant process?. Nephrol Dial Transplant.

[14] Lindroos M, Kupari M, Heikkilä J, Tilvis R (1993). Prevalence of aortic valve abnormalities in the elderly: an echocardiographic study of a random population sample. J Am Coll Cardiol.

[15] Iung B, Baron G, Butchart EG, Delahaye F, Gohlke-Bärwolf C, Levang OW, Tornos P, Vanoverschelde JL, Vermeer F, Boersma E, Ravaud P, Vahanian A (2003). A prospective survey of patients with valvular heart disease in Europe: the euro heart survey on valvular heart disease. Eur Heart J.

[16] Dweck MR, Boon NA, Newby DE (2012). Calcific aortic stenosis: a disease of the valve and the myocardium. J Am Coll Cardiol.

[17] Rajamannan NM, Evans FJ, Aikawa E, Grande-Allen KJ, Demer LL, Heistad DD, Simmons CA, Masters KS, Mathieu P, O'Brien KD, Schoen FJ, Towler DA, Yoganathan AP, Otto CM (2011). Calcific aortic valve disease: not simply a degenerative process: A review and agenda for research from the National Heart and Lung and Blood Institute Aortic Stenosis Working Group. Executive summary: Calcific aortic valve disease-2011 update. Circulation.

[18] Rabkin SW (2005). The association of hypertension and aortic valve sclerosis. Blood Press.

[19] Cowell SJ, Newby DE, Prescott RJ, Bloomfield P, Reid J, Northridge DB, Boon NA, Scottish Aortic Stenosis and Lipid Lowering Trial, Impact on Regression (SALTIRE) Investigators (2005). A randomized trial of intensive lipid-lowering therapy in calcific aortic stenosis. N Engl J Med.

[20] Rossebø AB, Pedersen TR, Boman K, Brudi P, Chambers JB, Egstrup K, Gerdts E, Gohlke-Bärwolf C, Holme I, Kesäniemi YA, Malbecq W, Nienaber CA, Ray S, Skjaerpe T, Wachtell K, Willenheimer R, SEAS Investigators (2008). Intensive lipid lowering with simvastatin and ezetimibe in aortic stenosis. N Engl J Med.

[21] Novaro GM, Katz R, Aviles RJ, Gottdiener JS, Cushman M, Psaty BM, Otto CM, Griffin BP (2007). Clinical factors, but not C-reactive protein, predict progression of calcific aortic-valve disease: the cardiovascular health study. J Am Coll Cardiol.

[22] Bansal N, McCulloch CE, Lin F, Robinson-Cohen C, Rahman M, Kusek JW, Anderson AH, Xie D, Townsend RR, Lora CM, Wright J, Go AS, Ojo A, Alper A, Lustigova E, Cuevas M, Kallem R, Hsu CY, CRIC Study Investigators (2016). Different components of blood pressure are associated with increased risk of atherosclerotic cardiovascular disease versus heart failure in advanced chronic kidney disease. Kidney Int.

[23] Cheung AK, Rahman M, Reboussin DM, Craven TE, Greene T, Kimmel PL, Cushman WC, Hawfield AT, Johnson KC, Lewis CE, Oparil S, Rocco MV, Sink KM, Whelton PK, Wright JT, Basile J, Beddhu S, Bhatt U, Chang TI, Chertow GM, Chonchol M, Freedman BI, Haley W, Ix JH, Katz LA, Killeen AA, Papademetriou V, Ricardo AC, Servilla K, Wall B, Wolfgram D, Yee J, SPRINT Research Group (2017). Effects of Intensive BP control in CKD. J Am Soc Nephrol.

[24] Haffner SM, Lehto S, Rönnemaa T, Pyörälä K, Laakso M (1998). Mortality from coronary heart disease in subjects with type 2 diabetes and in nondiabetic subjects with and without prior myocardial infarction. N Engl J Med.

[25] Tonelli M, Muntner P, Lloyd A, Manns BJ, Klarenbach S, Pannu N, James MT, Hemmelgarn BR, Alberta Kidney Disease Network (2012). Risk of coronary events in people with chronic kidney disease compared with those with diabetes: a population-level cohort study. Lancet.

[26] Adler AI, Stevens RJ, Manley SE, Bilous RW, Cull CA, Holman RR, UKPDS GROUP (2003). Development and progression of nephropathy in type 2 diabetes: the United Kingdom Prospective Diabetes Study (UKPDS 64). Kidney Int.

[27] Kestenbaum B, Sampson JN, Rudser KD, Patterson DJ, Seliger SL, Young B, Sherrard DJ, Andress DL (2005). Serum phosphate levels and mortality risk among people with chronic kidney disease. J Am Soc Nephrol.

[28] Tentori F, Blayney MJ, Albert JM, Gillespie BW, Kerr PG, Bommer J, Young EW, Akizawa T, Akiba T, Pisoni RL, Robinson BM, Port FK (2008). Mortality risk for dialysis patients with different levels of serum calcium, phosphorus, and PTH: the Dialysis Outcomes and Practice Patterns Study (DOPPS). Am J Kidney Dis.

[29] Giachelli CM, Speer MY, Li X, Rajachar RM, Yang H (2005). Regulation of vascular calcification: roles of phosphate and osteopontin. Circ Res.

[30] Shroff R, Long DA, Shanahan C (2013). Mechanistic insights into vascular calcification in CKD. J Am Soc Nephrol.

[31] Goodman WG, Goldin J, Kuizon BD, Yoon C, Gales B, Sider D, Wang Y, Chung J, Emerick A, Greaser L, Elashoff RM, Salusky IB (2000). Coronary-artery calcification in young adults with end-stage renal disease who are undergoing dialysis. N Engl J Med.

[32] Isaka Y, Hamano T, Fujii H, Tsujimoto Y, Koiwa F, Sakaguchi Y, Tanaka R, Tomiyama N, Tatsugami F, Teramukai S (2021). Optimal phosphate control related to coronary artery calcification in dialysis patients. J Am Soc Nephrol.

[33] Block GA, Klassen PS, Lazarus JM, Ofsthun N, Lowrie EG, Chertow GM (2004). Mineral metabolism, mortality, and morbidity in maintenance hemodialysis. J Am Soc Nephrol.

[34] Floege J, Kim J, Ireland E, Chazot C, Drueke T, de Francisco A, Kronenberg F, Marcelli D, Passlick-Deetjen J, Schernthaner G, Fouqueray B, Wheeler DC, ARO Investigators (2011). Serum iPTH, calcium and phosphate, and the risk of mortality in a European haemodialysis population. Nephrol Dial Transplant.

[35] Ganesh SK, Stack AG, Levin NW, Hulbert-Shearon T, Port FK (2001). Association of elevated serum PO(4), Ca x PO(4) product, and parathyroid hormone with cardiac mortality risk in chronic hemodialysis patients. J Am Soc Nephrol.

[36] Young EW, Albert JM, Satayathum S, Goodkin DA, Pisoni RL, Akiba T, Akizawa T, Kurokawa K, Bommer J, Piera L, Port FK (2005). Predictors and consequences of altered mineral metabolism: the dialysis outcomes and practice patterns study. Kidney Int.

[37] Slinin Y, Foley RN, Collins AJ (2005). Calcium, phosphorus, parathyroid hormone, and cardiovascular disease in hemodialysis patients: the USRDS waves 1, 3, and 4 study. J Am Soc Nephrol.

[38] Rattazzi M, Bertacco E, Del Vecchio A, Puato M, Faggin E, Pauletto P (2013). Aortic valve calcification in chronic kidney disease. Nephrol Dial Transplant.

[39] El Husseini D, Boulanger MC, Fournier D, Mahmut A, Bossé Y, Pibarot P, Mathieu P (2013). High expression of the Pi-transporter SLC20A1/Pit1 in calcific aortic valve disease promotes mineralization through regulation of Akt-1. PLoS One.

[40] Tarrass F, Benjelloun M, Zamd M, Medkouri G, Hachim K, Benghanem MG, Ramdani B (2006). Heart valve calcifications in patients with end-stage renal disease: analysis for risk factors. Nephrology (Carlton).

[41] Petrović D, Obrenović R, Stojimirović B (2009). Risk factors for aortic valve calcification in patients on regular hemodialysis. Int J Artif Organs.

[42] Kalantar-Zadeh K, Regidor DL, Kovesdy CP, Van Wyck D, Bunnapradist S, Horwich TB, Fonarow GC (2009). Fluid retention is associated with cardiovascular mortality in patients undergoing long-term hemodialysis. Circulation.

[43] Cozzolino M, Mangano M, Stucchi A, Ciceri P, Conte F, Galassi A (2018). Cardiovascular disease in dialysis patients. Nephrol Dial Transplant.

[44] Sun J, Axelsson J, Machowska A, Heimbürger O, Bárány P, Lindholm B, Lindström K, Stenvinkel P, Qureshi AR (2016). Biomarkers of cardiovascular disease and mortality risk in patients with advanced CKD. Clin J Am Soc Nephrol.

[45] Liabeuf S, Lenglet A, Desjardins L, Neirynck N, Glorieux G, Lemke HD, Vanholder R, Diouf M, Choukroun G, Massy ZA, European Uremic Toxin Work Group (EUTox) (2012). Plasma beta-2 microglobulin is associated with cardiovascular disease in uremic patients. Kidney Int.

[46] Stubbs JR, House JA, Ocque AJ, Zhang S, Johnson C, Kimber C, Schmidt K, Gupta A, Wetmore JB, Nolin TD, Spertus JA, Yu AS (2016). Serum Trimethylamine-N-Oxide is elevated in CKD and correlates with coronary atherosclerosis burden. J Am Soc Nephrol.

[47] Barreto FC, Barreto DV, Liabeuf S, Meert N, Glorieux G, Temmar M, Choukroun G, Vanholder R, Massy ZA, European Uremic Toxin Work Group (EUTox) (2009). Serum indoxyl sulfate is associated with vascular disease and mortality in chronic kidney disease patients. Clin J Am Soc Nephrol.

